# ColdZyme® protects airway epithelia from infection with BA.4/5

**DOI:** 10.1186/s12931-022-02223-2

**Published:** 2022-10-31

**Authors:** Zaderer Viktoria, Dichtl Stefanie, Bellmann-Weiler Rosa, Lass-Flörl Cornelia, Posch Wilfried, Wilflingseder Doris

**Affiliations:** 1 Institute of Hygiene and Medical Microbiology, Medical University of Innsbruck, Schöpfstrasse 41/R311, 6020 Innsbruck, Austria; 2 Department of Internal Medicine II, Medical University of Innsbruck, Innsbruck, Austria

**Keywords:** SARS-CoV-2, Prophylaxis, Variants of concern, BA.4, BA.5, Transmission

## Abstract

Vaccines against SARS-CoV-2 protect from critical or severe pathogenesis also against new variants of concern (VOCs) such as BA.4 and BA.5, but immediate interventions to avoid viral transmission and subsequent inflammatory reactions are needed. Here we applied the ColdZyme® medical device mouth spray to fully differentiated, polarized human epithelium cultured at an air-liquid interphase (ALI). We found using VOCs BA.1 and BA.4/5 that this device effectively blocked respiratory tissue infection. While infection with these VOCs resulted in intracellular complement activation, thus enhanced inflammation, and drop of transepithelial resistance, these phenomena were prevented by a single administration of this medical device. Thus, ColdZyme® mouth spray significantly shields epithelial integrity, hinders virus infection and blocks in a secondary effect intrinsic complement activation within airway cultures also in terms of the highly contagious VOCs BA.4/5. Crucially, our in vitro data suggest that ColdZyme® mouth spray may have an impact to protect against SARS-CoV-2 transmission, also in case of the Omicron BA.1, BA.4 and BA.5 variants.

## Introduction

Novel SARS-CoV-2 variants of concern (VOC) rapidly emerge. In January 2022 the two recent Omicron lineages, BA.4 and BA.5 (from here on referred to as BA.4/5), appeared in South Africa [1]. BA.4/5 has rapidly replaced BA.1 and BA.2 and caused a new COVID-19 wave in Europe in early summer 2022, due to a better transmissibility and higher infectivity relative to BA.2 [1]. BA.4/5 have identical viral spike (S) protein sequences. BA.4/5 contains additional mutations in the receptor-binding domain (RBD), in particular mutations 69-70del, L452R and F486V, while it is lacking the R493Q substitution relative to BA.2 [1, 2]. Currently used vaccines are designed against wildtype SARS-CoV-2. Due to its multiple mutations, the SARS-CoV-2 Omicron sublineage was reported to escape from vaccine- and infection-elicited antibodies [3–8], making these VOCs highly transmissive causing high incident rates. Thus, even after more than two years, still, up to date efficient treatments to avoid the transfer of the virus from one to another are lacking.

As recently shown, ColdZyme® mouth spray effectively blocked binding, uptake and infection of SARS-CoV-2 wildtype in a human respiratory model that was associated with rescue to tissue integrity and from excessive innate immune activation [9]. ColdZyme® is a Class III medical device (CE-marked) composed of glycerol, water, buffer, CaCl2, menthol and trypsin from the Atlantic cod (*Gadus morhua*) [10] (ClinicalTrials.gov, ID: NCT03901846). The ColdZyme® mouth spray forms a physical barrier that interferes with entry of common cold viruses, which subsequently become trapped and inactivated [11, 12]. Due to these actions, viral entry might be hampered by ColdZyme® and this was illustrated in vitro using viruses such as rhinovirus, RSV or influenza [11]. ColdZyme inactivated these viruses in a range between 60 and 100%, when applied for 20 min at 35 to 37 °C [11]. Beside its high effectiveness, the medical device also exerts a high safety profile, which can be explained by high sensitivity of Cod trypsin, an ingredient of ColdZyme®, to pH and heat within the oral mucosa. Beside rhinovirus, RSV and influenza, SARS-CoV-2 as well as the common cold coronavirus HCoV-229E were demonstrated to be sensitive to prophylactic treatment with ColdZyme® mouth spray in previous in vitro analyses within various cellular systems, i.e. Vero E6 and MRC-5 cells, but also in 3D human airway epithelial tissue cultures [9, 13]. Posch et al. recently showed a robust engagement of complement in human airway epithelial (HAE) tissue cultures after viral infection, which was associated with high anaphylatoxin and pro-inflammatory cytokine release, high viral loads and excessive tissue destruction [9, 13]. These virus-mediated effects could be efficiently interferred with by blocking the anaphylatoxin receptor C5aR at basolateral sites of epithelial tissues, but also by applying the ColdZyme® mouth spray to the highly differentiated primary nasal and bronchial epithelial 3D tissue models prior infection with SARS-CoV-2 wildtype virus [9, 13]. Thus, we, here, tested the efficacy of the spray against the highly contagious Omicron variants BA.1, and more importantly, BA.4/5. While tissue destruction with concomitant innate immune, complement C3, activation and high release of viruses into the basolateral subnatant were detected upon infection with BA.1 and BA.4/5, tissue integrity was completely restored and local complement C3 production and virus release blocked by the ColdZyme® mouth spray. Our results point towards an easy-to-use regularly, prophylactic treatment using ColdZyme® mouth spray in order to prevent infection in particular with the current, highly transmissible BA.4/5 variants.

## Materials and methods

### Ethics statement

Written informed consent was obtained from all donors of leftover nasopharyngeal/oropharyngeal specimens and EDTA blood by the participating clinics. The Ethics Committee of the Medical University of Innsbruck (ECS1166/2020) approved the use of anonymized leftover specimens of COVID-19 patients for scientific purposes.

### Cell culture of tissue models and ColdZyme® treatment

#### HAE

Normal human bronchial epithelial (NHBE, Lonza, cat# CC-2540 S) are available in our laboratary and routinely cultured in air liquid interface (ALI) as described [14, 15]. Briefly, cells were cultured in a T75 flask for 2–4 days until they reached 80% confluence. The cells were trypsinized and seeded onto GrowDexT (UPM)-coated 0.33 cm^2^ porous (0.4 μm) polyester membrane inserts with a seeding density of 1 × 10^5^ cells per Transwell (Costar, Corning, New York, NY, USA). The cells were grown to near confluence in submerged culture for 2–3 days in specific epithelial cell growth medium according to the manufacturer´s instructions. Cultures were maintained in a humidified atmosphere with 5% CO2 at 37 °C and then transferred to ALI culture. The epithelium was expanded and differentiated using airway media from Stemcell™. The number of days in development was designated relative to initiation of ALI culture, corresponding to day 0. One hub of ColdZyme® mouthspray was applied to the apical side of the fully differentiated epithelia prior to infection using BA0.1 or BA0.4/5. This corresponded to approximately 50 µl of liquid, evenly dispersed over the tissue culture. The apical application was carefully performed to not mechanically disrupt the epithelial surface.

#### Vero cells

VeroE6/TMPRSS2/ACE2 is an engineered VeroE6 cell line expressing high levels of TMPRSS2 and ACE2 and highly susceptible to SARS-CoV-2 infection. This cell line was used to expand characterized BA.1 and BA.4/5 viruses from patient isolates. The cell line was obtained via the CFAR (NIBSC) and is described in [16].

### TEER measurement

TEER values were measured using EVOM volt-ohm-meter with STX-2 chopstick electrodes (World Precision Instruments, Stevenage, UK). Measurements on cells in ALI culture were taken immediately before the medium was exchanged. For measurements, 0.1 ml and 0.7 ml of medium were added to the apical and basolateral chambers, respectively. Cells were allowed to equilibrate before TEER was measured. TEER values reported were corrected for the resistance and surface area of the Transwell filters.

### Staining and high content screening (HCS)

To visualize SARS-CoV-2 infection in monolayers and 3D tissue models, cells were infected with clinical specimen of SARS-CoV-2 and analyzed for characteristic markers in infection experiments on day 3 post infection (3 dpi). After SARS-CoV-2 exposure, 3D cell cultures were fixed with 4% paraformaldehyde. Intracellular staining was performed using 1× Intracellular Staining Permeabilization Wash Buffer (10X; BioLegend, San Diego, CA, USA). The cell surface was stained using WGA (wheat germ agglutinin)-680, Thermofisher Scientific, Waltham, MA, USA), nuclei using Hoechst 33,342, Cell Signaling Technologies, Danvers, MA, USA, and complement C3 (C3-FITC, Agilent Technologies, Santa Clara, CA, USA ) were used. Intracellular SARS-CoV-2 was detected using Alexa594-labeled SARS-CoV-2 antibodies against S1 and N (both Sino Biological, Beijing, China). The Alexa594-labeling kit was purchased from Abcam, Cambridge, UK. After staining, 3D cultures were mounted in Mowiol. To study these complex models using primary cell cultured in 3D and to generate detailed phenotypic fingerprints for deeper biological insights in a high throughput manner, the Operetta CLS System (PerkinElmer, Waltham, MA, USA) was applied. Spot analyses and absolute quantification for SARS-CoV-2-containing cells, Harmony™ Software was performed in more than 1200 cells per condition, since in three independent experiments at least 400 cells defined by nuclear stain (Höchst) were analyzed.

### Viruses

Clinical specimens for SARS-CoV-2 Omicron BA.1 and BA.4/5 from COVID-19 positive swabs, sequenced by the Austrian Agency for Health and Food Safety, Vienna, Austria (Ethics statement, ECS1166/2020) were propagated and used subsequently to infect cells.

### Statistical analysis

Statistical analysis of differences in infection levels, TEER values, or cytokine production was performed utilising the GraphPad prism software and using OneWay ANOVA with Tukey´s post test.

## Results

### ColdZyme® mouth spray protects from BA.1 and BA.4/5 infection and intracellular (IC) C3 activation in primary human bronchial epithelial (NHBE) cells

In first experiments, we monitored primary normal human bronchial epithelial (NHBE) cell infection using BA.1 as well as the recent VOCs BA.4/5 in absence and presence of the ColdZyme® mouth spray. For infection assays, a multiplicity of infection (MOI) of 0.01 was used to determine morphogenesis and cytopathic effects of SARS-CoV-2 infection in human airway epithelial cells.

**Fig. 1:**
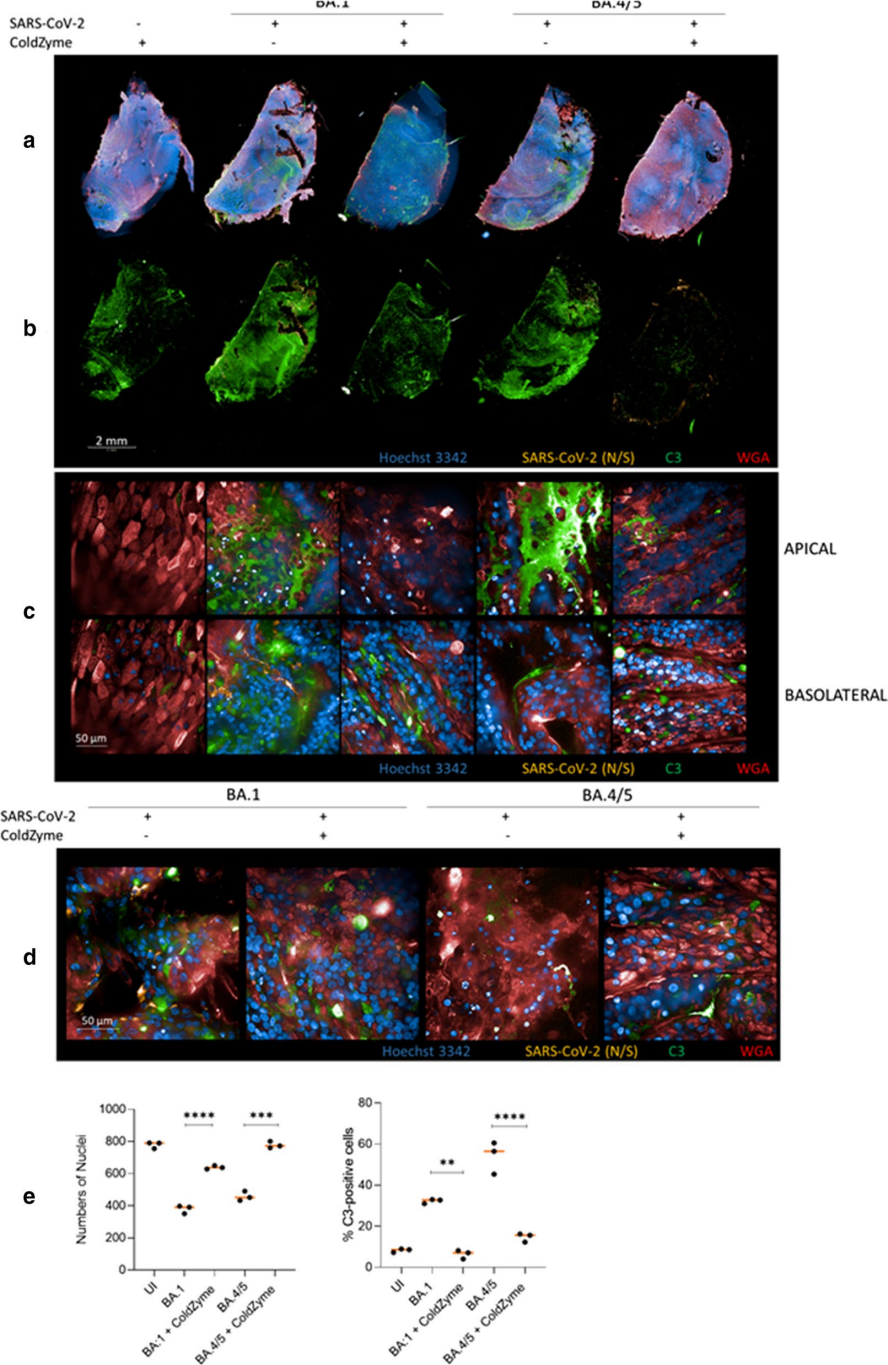
ColdZyme® mouth spray protects primary human airway epithelial (HAE) cells from SARS-CoV-2 BA.1 and BA.4/5 infection and innate immune activation. Visualization of virus binding (SARS-CoV-2 S1/N, orange) and complement (C3-FITC, green) in SARS-CoV-2 infected 3D pseudostratified epithelia. Pseudostratified epithelia were apically treated with ColdZyme® mouth spray prior exposure to SARS-CoV-2 (MOI 0.01). On 3 dpi, filters were fixed, stained for höchst (blue), SARS-CoV-2 S1/N (orange), complement C3 (green) and WGA (red) and then analysed by HCS. **a** Overview on one half of a Transwell filter of solvent/ColdZyme®-controls (panel 1), BA0.1- or BA0.4/5-infected (panel 2 and 4), ColdZyme-pre-treated and BA0.1 or BA0.4/5-infected (panel 3 and 5) HAE cultures using the 5X magnification and showing all markers or **b** complement C3 (green) and SARS-CoV-2 (N/S) (orange) markers. One representative filter half is illustrated. Scale bars represent 2 mm. **c** Z-stacks of uninfected (UI, panel 1), BA.1- or BA.4/5-infected (panel 2 and 4), ColdZyme-pre-treated and BA.1 or BA.4/5-infected (panel 3 and 5) HAE cultures were analyzed using the Operetta CLS HCS and the 63XWATER objective. Cells were stained using C3-FITC (green) as indicator for innate immune activation, SARS-CoV-2-S1/N-Alexa594 (orange) for virus detection, höchst for imaging nuclei (blue) and WGA for staining lectins (red). High IC C3 mobilization was monitored in BA.1- and BA.4/5-infected cultures, while no virus and low C3 signals were detected in UI (panel 1) and ColdZyme®/BA.1 or BA.4/5- (panel 3 and 5) cultures. Scale bars represent 50 μm and three independent experiments were performed. **d** Tissue destruction upon viral infection (BA0.1, panel 1; BA0.4/5, panel 3) and rescue by single ColdZyme® application prior infection (BA.1, panel 2; BA.4/5, panel 4) are shown. Scale bars represent 50 μm and three independent experiments were performed. **e** More than 1000 cells per condition (UI, BA.1, BA.1 + ColdZyme), BA.4/5, BA.4/5 + ColdZyme) were analyzed for their expression of C3, where up to 38% for BA.1 and 60% for BA.4/5 of the analyzed cells were stained positive for C3. Only 10–18% of C3 signal were detected in UI or ColdZyme/BA.1 or BA.4/5-exposed cells. Statistical significances were analyzed with GraphPad Prism software using One-way ANOVA and Tukey´s post test

We recently found that infection in primary airway epithelial cells was accompanied by extensive induction of intracellular (IC) C3 and secretion of the anaphylatoxin C3a from HAE cells [13]. Thus, we used IC C3 as an indicator for innate immune activation during infection of NHBE cells with BA.1 and BA.4/5.

**Fig. 2:**
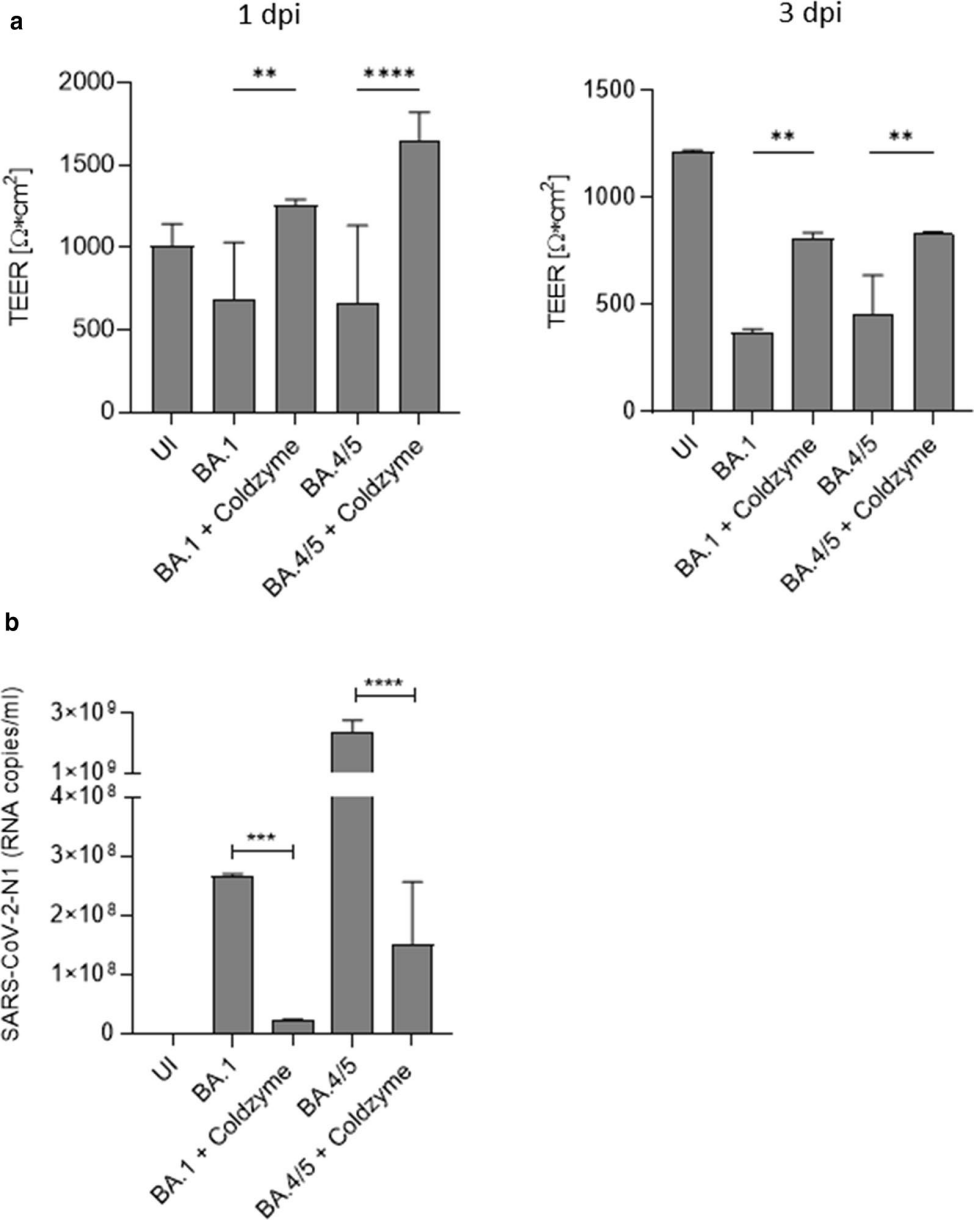
Disruption of epithelial integrity by BA.1 and BA.4/5 can be avoided by ColdZyme® pre-treatment. **a** Pseudostratified epithelia were infected by apical addition of SARS-CoV-2 BA.1 and BA.4/5 (MOI 0.01) with or without ColdZyme® pretreatment and incubated for 72 h. TEER was measured on 1 dpi (left) and 3 dpi (right) using a EVOM volt-ohm-meter. TEER in Ω/cm^2^ was determined for all conditions (UI, BA.1, BA.1 + ColdZyme, BA.4/5, BA.4/5 + ColdZyme) and plotted on a bar graph. Bars represent the mean + SD from 3 independent pseudostratified epithelia measured in triplicates. Statistical significance was calculated using One-way ANOVA with Tukey´s multiple comparisons test. **b** Viral RNA was measured from UI, BA.1, BA.1 + ColdZyme, BA.4/5, and BA.4/5 + ColdZyme cultures of pseudostratified epithelia on 3 dpi. The experiment was repeated at least 3 times and statistically significantl differences were determined by one-way ANOVA with Tukey´s multiple comparisons test. All values are means ± SD

Imaging the overview of mock-treated and BA.1- or BA.4/5-infected NHBE cells pre-treated with ColdZyme® mouth spray already revealed the intactness of the Transwell halves (Fig. 1a, 1st I 3rd I 5th panel), with nearly no virus in infected cultures (Fig. 1b, orange, 1st I 3rd I 5th panel) and little IC C3 activation (Fig. 1b, green, 1st I 3rd I 5th panel). In contrast, BA.1 (Figs. 1b and 2nd panel)- and BA.4/5 (Figs. 1b and 4th panel)-infected tissues displayed a productive virus infection (orange) going along with concomitant activation of IC C3 (green) (Figs. 1b, c and 2nd and 4th panels; Fig. 1d, 1st and 3rd panel).

Thus, not only BA.1 and BA.4/5-infection were hampered, if tissues were pre-treated with the ColdZyme® mouth spray, but the epithelia were also rescued from IC C3 mobilization and protected from infection and subsequent tissue damage (Fig. 1b–d) as revealed in more detail by imaging higher magnification of the tissues (Fig. 1c, d). In Fig. 1c, excessive IC C3 activation throughout the epithelium was observed upon infection with BA.1 (2nd panel) and BA.4/5 (4th panel), while C3 signals were minor in UI (1st panel), ColdZyme®/BA.1 (3rd panel) and ColdZyme®/BA.4/5-exposed upper respiratory tract tissues (Fig. 1c, green). Moreover, tissue integrity was completely protected in ColdZyme®-pretreated tissues independent on the VOC used (Fig. 1d).

Since IC C3 levels in treated and infected cultures were comparable to those of ColdZyme®-treated, uninfected samples (Fig. 1a–c, left), next, we analyzed and quantified C3 signals from more than 1000 cells/condition in UI, BA.1 and BA.4/5- and ColdZyme®/BA.1 and /BA.4/5-exposed, pseudostratified respiratory epithelia. We found highly significant differences in cell numbers and percentages of C3^+^- (Fig. 1e) cells between cultures infected with the two SARS-CoV-2-VOCs compared to ColdZyme-treated/uninfected and ColdZyme®-treated/SARS-CoV-2-VOC-infected, primary respiratory cultures. These analyses demonstrate that infection going along with tissue destruction and intracellular C3 mobilization on 3 dpi induced in NHBE cultures upon SARS-CoV-2 interactions can be avoided by pre-treatment of epithelia with ColdZyme® mouth spray.

### ColdZyme® mouth spray maintains epithelial integrity upon SARS-CoV-2 infection of NHBE cultures

To monitor, if ColdZyme® mouth spray protects respiratory tissues from SARS-CoV-2-mediated destruction in long-term, NHBE cultures were kept in culture for 3 days. Analysis of transepithelial electrical resistance (TEER; Fig. 2a), an indicator for tissue integrity, was performed over time on 1 dpi and 3 dpi. These analyses revealed that upon BA.1- and BA.4/5 infection, TEER values dropped 1 dpi (left), though the drop was not as prominent as with earlier variants of SARS-CoV-2 [9, 13]. On 3 dpi, TEER values of infected cultures were significantly lower (right) (Fig. 2a) compared to controls or ColdZyme®-treated/infected cultures independent on the strain used (Fig. 2a, UI, BA.1 + ColdZyme, BA.4/5 + ColdZyme). While in ColdZyme-treated UI as well as infected tissue cultures TEER values ranged from 700 to 1600 Ω/cm^2^, this range dropped to 250 to 650 Ω/cm^2^ in BA.1 and BA.4/5-infected cells (Fig. 2a, left and right). Tissue integrity was greatly rescued in infection experiments on both days analyzed post infection, if ColdZyme® was applied prior SARS-CoV-2 exposure only once. These analyses are in accordance with the immunofluorescence data, illustrating enhanced destruction of superficial, pseudostratified epithelial layers by Omicron VOCs BA.1 and BA.4/5 on 3 dpi, while prophylactic treatment with ColdZyme® mouth spray greatly prevented tissues from this virus-mediated destruction.

### Viral loads are significantly decreased by prophylactic ColdZyme® mouth spray application

In accordance to image analyses and TEER, absolute quantification of viral load from 3 dpi subnatants of differently treated cells revealed protection from infection by applying the ColdZyme® mouth spray (Fig. 2b). Viral loads in differentially treated tissues were determined by quantitative real-time RT-PCR using a SARS-CoV2-2-N1 standard. SARS-CoV-2-infected cultures illustrated productive infection with BA.1 (mean ~ 2.8*10^8^ copies/ml) and BA.4/5 (mean ~ 2.8*10^9^ copies/ml), which was significantly reduced by one-time application of ColdZyme® mouth spray for both VOCs (Fig. 2b). Here, we found that single application of ColdZyme® mouth spray blocked SARS-CoV-2 infection of HAE cultures not only in terms of protecting tissue integrity and inhibiting innate immune activation, and finally by significantly reducing virus release.

## Discussion

Here, we found that one hub of ColdZyme® mouth spray was sufficient to block Omicron BA.4/5 and BA.1 from infecting highly differentiated, mucus-producing and ciliated primary human bronchial airway epithelial tissue cultures. Since ColdZyme® was recently shown to avoid highly destructive and inflammatory effects exerted by SARS-CoV-2 wildtype virus upon interaction with pseudostratified epithelia [9] and due to the high transmissibility of BA.4/5 despite vaccination, easy-to-use products locally effective at mucosal sites, where the virus enters, are still desperately needed.

The ColdZyme® mouth spray forms a physical barrier interfering with entry of common cold viruses and a higher inactivation capacity for enveloped viruses RSV and influenza compared to non-enveloped rhinoviruses was described [11, 12]. The oral mucosa itself is very well protected against proteolytic enzymes such as trypsin, an ingredient of ColdZyme®, by protease inhibitors and mucins, and cod trypsin is more sensititve to pH and heat, thereby explaining the high safety profile of the ColdZyme® mouth spray [11]. Moreover, it was illustrated that cod trypsin has higher catalytic efficiency than comparable enzymes [11]. As well, cod trypsin appears to be very effective in hydrolyzing native proteins [17]. Cod trypsin production involves extraction, filtration and chromatography steps conducted in an environmentally friendly manner using byproducts of Atlantic cod (Gadus morhua) [11, 17]. We earlier observed that the protective effect from SARS-CoV-2 wildtype infection in vitro was up to 2 h following application of the ColdZyme® mouth spray to the apical side of fully differentiated respiratory epithelia [9]. A clinical trial on evaluation of ColdZyme® to experimentally induced common cold applied 6 doses of the mouth spray daily, which corresponds to our time window of 1 to 2 h effectiveness described earlier (clinical trial: COLDPREVII, https://clinicaltrials.gov/ct2/show/NCT02479750).

Since nasal and oral epithelium are portals for initial infection and transmission, and Omicron VOCs (BA.1, BA.2, BA.4, BA.5) are highly contagious, in our study, ColdZyme® mouth spray was applied to normal human bronchial epithelial of the upper respiratory tract grown in air-liquid interphase. Comparable to studies of Vero E6 and MRC-5 monolayers or HAE tissue models on the efficacy of ColdZyme® mouth spray against SARS-CoV-2 wildtype [9], we here describe an efficient inactivation of BA.1 as well as BA.4/5 by distributing ColdZyme® in a single application to highly differentiated tissues. BA.1 and BA.4/5 that productively infected HAE tissues were efficiently inactivated by one hub of ColdZyme® and the effect lasted for 3 days. This is noteworthy, since within our in vitro model the virus cannot be removed by mucociliary clearance due to the design of the Transwell filter with plastic borders on each side and due to the static nature of such systems. The mouth spray contains glycerol as well as the above-described trypsin from the Atlantic cod, that together form a protective barrier [12]. The glycerol may play a role, due to its viscosity and high osmotic effect, in trapping of viral particles, where trypsin limits the ability of the virus to infect. Moreover, mucus production and elevated mucociliary clearance within the epithelia might be started due to applying the mouth spray, which we observed within ColdZyme®-treated epithelia applying fluorescently labeled beads and live cell analyses (not shown). Not only in case of SARS-CoV-2 wildtype, but also with the novel VOCs BA.1 and BA.4/5, we could not detect any virus deposition within the epithelial layers using the ColdZyme® mouth spray. Infection, tissue destruction and innate immune activation was not as prominent with the Omicron variants as earlier observed using previous versions [9, 13], but nevertheless a protective measure is indispensable to hinder the virus from entering our body and potentially decreasing the numbers of SARS-CoV-2 cases, which needs further proof.

Here, we describe that ColdZyme® completely antagonized BA.1 and BA.4/5 infection and concomitant virus-induced tissue damage and local complement activation as indicator for innate immune activation. Exacerbating of injury by local complement activation in the airway epithelium was shown for infection with SARS-CoV-2 during the first waves of the pandemic [18, 19]. Additionally, increased anaphylatoxin levels (C3a, C5a) in plasma and lung homogenates have been implicated in the pathogenesis of various lung conditions including cystic fibrosis and idiopathic pulmonary fibrosis [20, 21]. Elevated anaphylatoxin levels resulted in down-modulation of regulators of complement activation such as CD55 and CD46 and changes in injury markers on HAE cells [20]. As illustrated in our highly differentiated, pseudostratified 3D models, high levels of C3a were secreted from the airway epithelium upon interaction with SARS-CoV-2 [13]. This was significantly down-modulated as a secondary effect by applying ColdZyme® due to significantly reducing productive infection of respiratory tissues [9]. Since C3a was generated upon SARS-CoV-2 infection in respiratory tissues, intrinsic C3 generation is a good indicator for successive anaphylatoxin production at sites of infection. Despite the milder pathogenesis described with Omicron compared to SARS-CoV-2 earlier variants (rev. in [22]), within our system we observed an intrinsic complement C3 generation at infection sites within pseudostratified epithelia concomitant with – mostly superficial - tissue destruction and virus release. These effects were halted in primary human airway epithelia by sole pre-treatment of epithelia with ColdZyme®. This not only resulted in protection of high transepithelial electrical resistance, a marker of epithelial integrity, but also in significantly lowering of intracellular C3 production and virus release.

In summary, we propose that the application of ColdZyme® mouth spray represents a simple and promising approach not only in SARS-CoV-2 wildtype prevention or reduction in SARS-CoV-2-related symptoms by blocking virus binding and subsequent infection as well as protecting from complement activation and tissue damage as a secondary effect, but also against the highly ontagious variants BA.4/5 and BA.1. Although the results from our realistic in vitro HAE models are not directly translatable into in vivo efficacy, they open up the exciting possibility that ColdZyme® can be applied in the prevention of contagious SARS-CoV-2 VOC transmission and spread.

## Data Availability

All data generated or analysed during this study are included in this article.
