# Correction: ColdZyme® protects airway epithelia from infection with BA.4/5

**DOI:** 10.1186/s12931-023-02326-4

**Published:** 2023-01-24

**Authors:** Viktoria Zaderer, Stefanie Dichtl, Rosa Bellmann-Weiler, Cornelia Lass-Flörl, Wilfried Posch, Doris Wilflingseder

**Affiliations:** 1 Institute of Hygiene and Medical Microbiology, Medical University of Innsbruck, Schöpfstrasse 41/R311, 6020 Innsbruck, Austria; 2 Department of Internal Medicine II, Medical University of Innsbruck, Innsbruck, Austria

**Correction: Respiratory Research (2022) 23:300** **https://doi.org/10.1186/s12931-022-02223-2**

Following publication of the original article [1], the authors identified an error that the given name and family name of all authors were erroneously transposed.

The incorrect citation is: Viktoria Z, Stefanie D, Rosa BW, Cornelia LF, Wilfried P, Doris W.

The correct citation is: Zaderer, V., Dichtl, S., Bellmann-Weiler, R., Lass-Flörl, C., Posch, W., Wilflingseder, D.

The author group has been updated above and the original article [1] has been corrected.
